# User-Centric Cell-Free Massive Multiple-Input-Multiple-Output System with Noisy Channel Gain Estimation and Line of Sight: A Beckmann Distribution Approach

**DOI:** 10.3390/e27030223

**Published:** 2025-02-21

**Authors:** Danilo B. T. Almeida, Marcelo S. Alencar, Wamberto J. L. Queiroz, Rafael M. Duarte, Francisco Madeiro

**Affiliations:** 1 Department of Electrical Engineering, Federal University of Campina Grande, Campina Grande 58429-900, Brazil; wamberto@dee.ufcg.edu.br; 2 Department of Electrical Engineering, Federal University of Paraíba, João Pessoa 58051-970, Brazil; malencar@iecom.org.br; 3 Department of Computer Engineering, Federal University of Vale do São Francisco, Petrolina 56304-917, Brazil; rafael.mouraduarte@univasf.edu.br; 4 Polytechnic School of Pernambuco, University of Pernambuco, Recife 50720-001, Brazil; madeiro@poli.br

**Keywords:** cell-free, channel capacity, distributed architecture, outage probability

## Abstract

This paper analyzes for the first time how the Beckmann distribution can be used to characterize the random variable that represents the envelope of the effective channel gain experienced by the *k*-th user equipment (UE) of a user-centric (UC) cell-free (CF) system in a scenario with noisy channel state information (CSI) estimation and line of sight (LoS). Additionally, it is shown how the Beckmann probability density function (PDF) can be used to derive the PDF and the cumulative density function (CDF) of the instantaneous signal-to-interference-plus-noise ratio (SINR) of the UC CF *k*-th UE, followed by applications in the ergodic capacity (EC) and outage probability (OP) expression derivations. It is shown that, regardless of the type of distribution considered for the channel gain between each access point (AP) and UE links, the effective gain presents a Beckmann distribution.

## 1. Introduction

The development of mobile devices stimulated the emergence of new services and functionalities that, in some way, require internet connectivity. The increased number of connected mobile devices and the new services that require high data transfer rates motivate the search for new software and hardware technologies for mobile communications. One of these options is the cell-free (CF) architecture, which eliminates the need to subdivide the space into cells, unlike current mobile cellular systems [1,2].

The CF systems are massive multiple-input-multiple-output (mMIMO) distributed systems, in which all access points (APs) serve all user equipment (UE) simultaneously. The short distance between UEs and APs, provided by the CF systems, is one of their major advantages [3]. It makes the experienced data transmission rates more uniform [1]. When the processing of the received signal is centralized in the central processing unit (CPU) and minimum mean-squared-error (MMSE) equalization is adopted, the spectral efficiency of CF systems surpasses that of mMIMO cellular systems [4].

In the CF scenario, the channel gain between each AP and UE is characterized by either Rayleigh or Rice distributions [1,2,4,5]. However, the downlink signal at each UE receiver input experiences an effective channel gain that is neither Rayleigh or Rice, and comes from the random signal variations that affect each downlink signal transmitted by the APs on the network. Therefore, the absence of a proper characterization of this effective channel gain is an impediment to the derivation of accurate metric expressions to assess the system performance and to distribute the network resources. This inaccurate characterization leads to the adoption of system bounds, such as achievable rate (AR), which is derived using Jensen’s inequality, for those purposes, as reported by [6,7,8,9,10,11,12,13,14,15,16,17,18,19,20,21,22].

The generalized chi-squared (GCS) distribution may be used to characterize the effective channel gain when all AP-to-UE links exhibit random variations described by the Rayleigh distribution, and perfectly estimated channel state information (CSI) is observed [23,24]. However, in scenarios with noisy CSI estimation, the GCS distribution does not describe the effective channel gain variations. Additionally, as the density of APs throughout the network coverage area increases, the UEs’ receiver may experience links in line of sight (LoS). As a consequence, the GCS distribution should not be considered anymore.

In this context, in the present paper, noisy CSI estimation and non-null LoS occurrence probability throughout the network coverage area are proposed to be taken into account to characterize the effective channel gain experienced by the downlink signal of the user’s receivers. According to the adopted approach, the Beckmann distribution naturally arises to characterize the effective channel gain and allows us to circumvent the GCS distribution limitations and derive expressions for outage probability (OP) and EC of a UC CF system.

Due to the channel gain estimation error, the Beckmann distribution arises when the Lyapunov Central Limit Theorem (LCLT) [25] is considered to characterize both the real and imaginary parts of the effective downlink channel gain by asymmetrical Gaussian random variables. Additionally, the real and imaginary parts of the multiuser interference experienced by the UEs are characterized, according to LCLT, by symmetrical Gaussian random variables. In addition to expressions of EC and OP, this paper also presents curves comparing the maximum transmission ratio upper bound, provided by Jensen’s inequality, with the EC for the Beckmann effective channel gain model.

The presented curves show a good adherence of the theoretical expression-based values to the simulated performance metric values, for effective Beckman fading with LoS and noisy CSI estimation, when compared to the GCS model.

### 1.1. Main Contributions

This paper focuses on the analysis of the impact of the estimation error of each AP-to-UE link CSI in the effective channel gain experienced by the downlink signal of the *k*-th UE, considering low-mobility UEs that randomly experience links in NLoS and LoS in a UC CF system.

In summary, the main contributions of the manuscript are as follows:

- Inclusion of the CSI estimation error in all AP-to-UE links: By taking into account the imperfect CSI estimation and by applying the LCLT, it is possible to characterize the resulting effective channel gain for all UE by the Beckmann distribution. To the best of the authors’ knowledge, this is the first time the effective channel gain is statistically characterized by the Beckmann distribution for noisy AP-to-UE channel gain estimation errors in CF scenarios;
- Derivation of expressions for the *k*-UE SNR PDF and CDF: After characterizing the UE effective channel gain by the Beckmann distribution, new expressions for the *k*-th UE SNR PDF and CDF are derived and applied to the derivation of the OP and the EC metrics. The latter exhibits a higher accuracy when compared with an AR expression derived using the Jensen inequality. It is worth mentioning that AR expressions are widely adopted in the literature due to its derivation simplicity;
- Analysis of the effect of adopting an inappropriate distribution to characterize the effective channel gain: It is shown, using Monte Carlo simulation, that assuming the inappropriate distribution to characterize the effective channel gain leads to incorrect metric value prediction and decreases the effectiveness of AP selection algorithms;
- Comparison between the simulated data with the theoretical values given by the derived expressions: It is shown that the simulated data present a good agreement with theoretical values predicted by the derived OP and EC expressions, even in scenarios with low numbers of APs and UEs.

### 1.2. Paper Outline

Briefly, this paper is organized as follows: In Section 2, related works are presented. The characteristics and parameters of the cell-free system are addressed in Section 3. Section 4 is dedicated to the signal received by the UEs in the downlink and the approach used. In Section 5, the SINR distribution is characterized. Section 6 presents the proposed outage probability and ergodic capacity expressions. Finally, Section 7, Section 8 and Section 9 present the simulation setup, the results and conclusions of the work, respectively.

### 1.3. Paper Notation

If *x* is a complex variable, Rex and Imx denote the real and imaginary components of *x*, respectively, and $x^{*}$ denotes its complex conjugate. If *x* is a complex Gaussian random variable, $x∼\mathrm{CN}\left(\mu_{x},\sigma_{x}^{2}\right)$ has a mean of $\mu_{x}$ and a variance of $\sigma_{x}^{2}/2$ per component. If *X* represents a set of elements, then #X denotes its cardinality.

## 2. Related Works

In most recent works on CF systems, subjects like equalization, pilot sequence assignment and AP clustering are evaluated by means of system bounds for metrics like spectral efficiency and AR. These bounds are derived considering the Jensen inequality and neglect the small-scale fading (SSF) effect. These expressions provide initial insights into the system behavior and serve as a guiding reference for algorithm developers.

The use of other equalization and precoding methods besides those based on maximum ratio has been evaluated since CF was proposed. The LSF decoder and variants of zero forcing and MMSE are examples [2,5,8,14,15,16]. In this work, we derive new closed-form expressions for UC CF systems assuming maximum-ratio transmission (MRT) precoding.

### 2.1. AP Selection Application

Variants of the CF systems have been proposed, with different forms of power control in data transmissions or pilot signals, resulting in improvements in system performance. In particular, several authors have focused on a variant of the CF, a concept known as a user-centric (UC) approach, in which each UE is served by a group of APs, chosen among all possible APs within the coverage area according to some decision criteria. In some works, large-scale fading (LSF) coefficients [7,8,9] between APs and UEs pairs, or the variances in the fading envelopes [10], were used as decision criteria to form UC clusters. Other works use the channel coefficient or its estimate [11,12,13]. An alternative approach is to use the signal-to-interference-plus-noise ratio (SINR) as an association criterion [24].

Sarker and Fapojuwo [21] proposed a method for associating UEs with APs in order to guarantee the system’s scalability. After association, a pilot sequence assignment method is applied. The proposed association method was called JUAP (Joint UE and AP Preference-based Association Scheme) and has two limitations: all UEs must be associated with some AP and a maximum number of UEs can be associated with each AP. Taking Greedy-based and Hungarian-based UE selection methods as a reference, the proposed method proved to be superior [21]. The criterion for choosing the combination of UEs and APs was the AR, which is an upper bound for the EC. However, opting for the AR over the EC may lead to a channel overestimation. Therefore, after a proper channel characterization, we derived input UE SINR and EC expressions, which are more accurate alternatives, rather than the AR one, for the purpose of AP selection.

A different system configuration, called cluster-centric, was proposed in [26]. In this configuration, groups of AP antennas are associated with clusters of UEs, and each user in the cluster is fairly associated with high-gain antennas. The authors considered zero-forcing (full and partial) processing on the downlink and uplink and used the UC CF system as a comparison parameter. Thus, taking the AR and achievable sum rates (ASRs), which consist of the sum of ARs, it was shown that, regardless of the number of users, the proposed system was able to provide greater capacity and fairness. The results obtained by the AR expression neglect the SCF effects that, in some way, may decrease the gain achieved in [26]. Therefore, we propose the channel characterization by the Beckmann distribution to circumvent the limitation imposed by omitting the SCF effects when deriving the AR expressions by applying Jensen’s inequality.

### 2.2. Channel Characterization

By using the GCS distribution, an AP selection algorithm, operating with an adaptive modulation technique, is proposed in [24]. The authors make use of the SINR per link as the assignment criterion for the UC CF approach. However, the SINR expression in [24] assumes that the effective channel gain is characterized by the GCS distribution, which considers purely non-LoS links and perfect CSI estimation. Hence, we propose to use the Beckmann distribution, to characterize the effective channel gain distribution, and adopt an AP selection algorithm similar to the one in [24], but taking into account a noisy CSI estimation and links in LoS.

As a way to assess the performance of the CF system under different conditions, and an attempt to support optimization algorithms, mathematical expressions are proposed for metrics and data flow analysis in backhaul [11,17]. Thus, for the sake of simplicity, AR expressions, derived using Jensen’s inequality, have been adopted by several authors to evaluate CF system performance and implement optimization procedures [18,19,20,21,22]. Since AR is an upper bound, it may not accurately reflect the true system capacity.

For instance, by considering the GCS distribution for the effective channel gain, an ergodic capacity (EC) expression was derived in [23] for a purely non-line-of-sight (NLoS) scenario with perfect CSI estimation. The derived expression performed better in predicting the real system performance when compared to AR expression in the literature. However, by considering the NLoS scenario and assuming perfect CSI estimation, the expressions may not be applied to a more generalized scenario, and this differs from our proposal. On the other hand, we propose to take noisy CSI estimation and links in LoS, characterizing the effective channel gain by the Beckmann distribution.

### 2.3. Optimization Application

An optimization problem for a hybrid indoor CF system, which combines radio frequency (RF) transmission with visible light communication (VLC) transmission, with the aim of maximizing the achievable sum rate (ASR), is formulated and solved in [27]. In this CF system model, VLC and RF access points cooperate to serve all users, who are equipped with photodetectors and antennas. The ASR consists of the sum of ARs of VLC and RF systems. The method works as follows: An adopted coefficient, which is linked to the signal transmitted by the observed AP, is changed in an exhaustive search until the ASR of the system’s users reaches the maximum. There is no direct power control, and the considered precoding method was zero-forcing. The ASR showed that, in systems with few UEs, VLC networks are more efficient than RF networks, but inferior to hybrid networks with the proposed allocation method. On the other hand, hybrid networks with random allocation of APs provided lower ASR than other systems, for different numbers of users. Regardless of the achieved performance gain with the proposed technique, the use of the ASR, derived from the AR expressions, suppresses the SCF information which may limit the performance gain achieved by the technique proposed in [27].

A simulator based on three-dimensional ray-tracing is proposed in [28]. In particular, the authors highlight the ability to simulate power amplifiers based on Rapp’s non-linear model. In the proposed cluster definition method, the Reference Signal Received Power (RSRP) between the UEs and the APs is the input to the cluster formation algorithm. The results show that a greater number of APs serving UEs improves the profile of the empirical cumulative density function (eCDF) curve for the average received signal strength (RSS) rate. According to the evolution of the curves, the improvement in performance tends to stop after an AP/UE of 6. Although ray-tracing simulators are valuable tools for the system design, it is worth mentioning that for real-time applications, EC and OP expressions, such as the ones we propose, may be preferable due to their abilities to predict the system state without the need for performing a usually time-consuming simulation. Additionally, for fast-varying channels, the OP value acts as a better metric than the RSRP, because, due to the channel’s nature, the observed RSRP value may be misinterpreted.

Regarding CF massive MIMO systems and vehicle-to-everything (V2X) networks operating in millimeter waves, Zhu et al. [29] developed a new joint optimization problem whose objective is to minimize interference in order to guarantee good spectral efficiency. An innovation in the work is the algorithm proposed to solve this problem: deep reinforcement learning (DRL)-based distributed double deep Q-network (D-DDQN). Assuming downlink transmission with conjugate beamforming, the authors formulated the problem based on the expression of the observed mobile terminal spectral efficiency. In this problem, each AP can serve up to *L* user devices; in addition, each downlink beam can only be allocated to a single user, and each UE is served by at least one AP. The Random UE assignment and the UE-center Greedy Competition Algorithm were used as benchmarks. The results show that the maximum virtual UE power (used to measure the level of interference) is lower for the proposed method. Despite using machine learning, it is worth noting that the entire problem was formulated based on the expression of spectral efficiency, which reveals the importance of quantifying this type of metric.

The aforementioned works reveal the importance of having more precise expressions for the system metrics, especially the transmission rates. Thus, we propose a different approach to obtaining EC and OP expressions of UC CF systems, in order to predict their performance more accurately and improve optimization processes.

## 3. System Characterization

**Figure 1:**
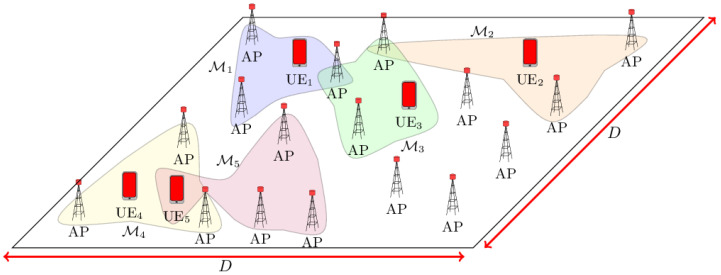
Representation of a UC CF system.

A UC CF system is assumed in which the *k*-th UE is supported by a set of APs, chosen among the *M* possible single-antenna APs within a D×D m^2^ coverage area, as shown in Figure 1.

The system operates according to a time division duplex (TDD) protocol, in which the frames are split into channel estimation, downlink payload data transmission and uplink payload data transmission, assuming channel gain reciprocity throughout the downlink and uplink (payload and training) phases [1].

The channel gain between the selected *m*-th AP and the *k*-th UE incorporates both the LSF and the uncorrelated flat SSF effects and can be expressed by

(1) $$g_{mk}=\sqrt{\beta_{mk}}\left(h_{mk}+{\bar{h}}_{mk}\right),$$

in which $\beta_{mk}$ takes into account the shadowing and the alpha–beta–gamma (ABG) path loss effect [24], ${\bar{h}}_{mk}$ denotes the AP-to-UE LoS component [30] and the NLoS channel component is characterized by $h_{mk}∼\mathrm{CN}\left(0,1\right)$ [1].

The SSF coefficient is considered frequency-non-selective and constant for a coherence time interval of $\tau_{s}$ symbols. In addition, considering a low-mobility scenario, the shadowing, which affects the average RSS, and the path loss, which is a function of the UE-to-AP distance, are assumed strongly time-correlated. Therefore, the LSF coefficient is considered to be constant for $\tau_{l}$ transmitted frames.

It is considered that, in the estimation phase, the CSI is acquired with error, so the estimated channel gain can be expressed as ${\hat{g}}_{mk}=g_{mk}+\epsilon_{mk}$, in which $\epsilon_{mk}$ is the channel gain estimation error. It is assumed that the MMSE estimator is used, and the estimation error may be characterized as $\epsilon_{mk}∼\mathrm{CN}\left(0,\sigma_{\epsilon_{mk}}^{2}\right)$, in which [31]

(2) $$\sigma_{\epsilon_{mk}}^{2}=\frac{\beta_{mk}}{1+\beta_{mk}\tau_{p}\rho_{p}},$$

with $\tau_{p}$ denoting the pilot sequence length and $\rho_{p}$ its transmission power.

## 4. Received Signal

If one defines $M_{k}$ and $K_{m}$ as the set of APs serving the *k*-th UE and the set of UEs being served by the *m*-th AP, respectively, the pre-coded downlink signal at the output of the matched filter of the *k*-th UE receiver may be expressed by [1]

(3) $$r_{k}=\sqrt{\rho }\sum_{m\in M_{k}}\sum_{j\in K_{m}}\eta_{mj}^{1/2}g_{mk}{\hat{g}}_{mj}^{*}q_{mj}+w_{k}=\alpha_{k}q_{k}+\psi_{k}+w_{k},$$

in which $w_{k}\in C$ represents the AWGN, ρ is the power per AP, $q_{mk}=q_{k}$ denotes the information symbol transmitted by the selected *m*-th AP to the *k*-th UE, with mean power $\sigma_{q}^{2}$,

(4) $$\alpha_{k}=\sqrt{\rho }\sum_{m\in M_{k}}\eta_{mk}^{1/2}g_{mk}{\hat{g}}_{mk}^{*}$$

is the effective channel gain,

(5) $$\psi_{k}=\sqrt{\rho }\sum_{m\in M_{k}}\sum_{\begin{array}{c} j\in K_{m}\\ j\neq k \end{array}}\eta_{mj}^{1/2}g_{mk}{\hat{g}}_{mj}^{*}q_{mj}$$

represents the multiuser interference and $\eta_{mk}$ denotes the power allocation coefficient.

It is worth mentioning that, regardless of whether each AP-to-UE channel gain $g_{mk}$ remains Rayleigh or Rice, the effective channel gain $\alpha_{k}$, given by the sum in (4), is characterized by the Beckmann distribution.

### 4.1. Effective Channel Gain Characterization

Due to the LSF, the elements in the sum that compose $\alpha_{k}$ in (4) are independent and non-identically distributed (INID). Hence, one can use the LCLT to characterize the real and the imaginary parts of $\alpha_{k}$ as Gaussian random variables [32].

The estimation error $\epsilon_{mk}$ depends on the used estimator’s structure and the noise power at the receiver. Therefore, it can be considered independent of the estimated signal. Thus, one can show that μImαk=0 and

(6) μReαk=ρ∑m∈Mkηmk1/2Egmk2=ρ∑m∈Mkηmk1/2βmk1+h¯mk2.

Additionally, one can demonstrate that

(7) σImαk2=ρ2∑m∈Mkηmkβmkσϵmk21+h¯mk2,

and

(8) σReαk2=ρ∑m∈Mkηmkβmk21+2h¯mk2+ρ2∑m∈Mkηmkβmkσϵmk21+h¯mk2.

The power imbalance of the real and imaginary components, expressed by the results in (7) and (8), and the real LoS component, quantified in (6), indicates the estimation error can introduce asymmetrical variations in the real and imaginary parts of the channel gain $\alpha_{k}$. Thus, to characterize the envelope of the effective channel gain $\left|\alpha_{k}\right|$, which presents asymmetrical Gaussian random variables components, it was necessary to consider the Beckmann distribution, which can be used to characterize the modulus of complex Gaussian random variables with arbitrary variances and arbitrary means. The Beckmann probability density function (PDF) is given by [33]

(9) pαkr=∫02πrexp−f(r,θ)2πσReαkσImαkdθ,

with

(10) fr,θ=rcosθ−μReαk22σReαk2+rsinθ−μImαk22σImαk2.

It is worth mentioning that, although the estimation error introduces a random variation in the imaginary component of the channel gain, giving rise to the Beckmann distribution, in scenarios with negligible $\epsilon_{mk}$, the effective channel gain distribution degenerates to the modulus of a non-zero-mean Gaussian random variable, a particular case of the Beckmann distribution. Therefore, if the *k*-th UE experiences AP-to-UE links in LoS, the GCS distribution is not appropriated to characterize the effective channel gain, but the Beckmann one is.

### 4.2. Multiuser Interference

Similarly, using the LCLT, the real and imaginary components of the multiuser interference $\psi_{k}$ can be characterized by Gaussian random variables.

Assuming $g_{mk}$, $g_{mj}$ and $q_{j}$ are mutually independent, $q_{mj}=q_{vj}$ ∀ *m* and *v* and that the real and imaginary components of $q_{j}$ are symmetrical, one can show that μReψk=μImψk=0. Unlike the effective channel gain, the multiuser interference exhibits symmetrical real and imaginary components. Therefore, one can show that σReψk2=σImψk2, with

(11) σReψk2=ρσq22∑m∈Mk∑v∈Mk∑j∈Kmj≠kBmvj,

in which, for m=v,

(12) $$B_{mmj}=\Delta_{mjk}\left(1+{\left|{\bar{h}}_{mk}\right|}^{2}\right)\left(1+{\left|{\bar{h}}_{mj}\right|}^{2}+\frac{\sigma_{\epsilon_{mj}}^{2}}{\beta_{mj}}\right)$$

and, for m≠v,

(13) $$B_{mvj}=\Delta_{mjk}^{1/2}\Delta_{vjk}^{1/2}\left(I_{mvk}+I_{mvj}\right),$$

with

(14) Imvu=Reh¯muReh¯vu+Imh¯mkImh¯vuReh¯muImh¯vu−Imh¯muReh¯vu−1,

and $\Delta_{mjk}=\eta_{mj}\beta_{mj}\beta_{mk}$.

## 5. SINR Distribution

The effective gain, introduced by $\alpha_{k}$, leads to a random variation in the SINR. Hence, after the matched filter of the *k*-th UE receiver, the observed SINR sample is

(15) $$\gamma_{k}={\left|\alpha_{k}\right|}^{2}\gamma_{qk},$$

in which $\gamma_{qk}$ denotes the per symbol SINR, given by

(16) $$\gamma_{qk}=\frac{\sigma_{q}^{2}}{\sigma_{\psi_{k}}^{2}+\sigma_{w_{k}}^{2}},$$

with

(17) σψk2=σReψk2+σImψk2

representing the total interference power experienced by the *k*-th UE.

By applying the variable transformation in (15) into (9) and adjusting the integration interval of the resulting Beckmann integral to (−1,1), one can use the Chebyshev–Gauss quadrature [34] to write the PDF of $\gamma_{k}$ as

(18) $$p_{\gamma_{k}}\left(y\right)=\frac{\left(1+\kappa_{k}\right)\left(1+\eta_{k}^{2}\right)}{2{\bar{\gamma }}_{k}N_{c}\eta_{k}\exp\left(p_{k}\right)}\sum_{c=1}^{N_{c}}\frac{\exp\left(r_{k}g_{c}\sqrt{y}\right)}{\exp\left[\upsilon_{k}\left(g_{c}\right)y\right]},$$

and the CDF of $\gamma_{k}$ as

(19) $$P_{\gamma_{k}}\left(y\right)=\frac{\left(1+\kappa_{k}\right)\left(1+\eta_{k}^{2}\right)}{2{\bar{\gamma }}_{k}N_{c}\eta_{k}\exp\left(p_{k}\right)}\sum_{c=1}^{N_{c}}\Psi \left(\upsilon_{k}\left(g_{c}\right),r_{k}g_{c},y\right),$$

in which

(20) $$p_{k}=\frac{\kappa_{k}}{2}\left(1+\frac{1}{\eta_{k}^{2}}\right)$$

and

(21) $$r_{k}=\frac{1+\eta_{k}^{2}}{\eta_{k}^{2}}\sqrt{\frac{\kappa_{k}\left(1+\kappa_{k}\right)}{{\bar{\gamma }}_{k}}}.$$

The Chebyshev–Gauss quadrature number of terms is denoted by $N_{c}$,

(22) $$g_{c}=\cos\left[\frac{\left(2c-1\right)}{N_{c}}\pi \right],$$

the real-to-imaginary component power ratio of the spread components of the effective channel gain fading is given by

(23) ηk2=σReαk2σImαk2,

with the normalized Rice factor given by

(24) κk=μReαk2σReαk2+σImαk2

and auxiliary functions

(25) $$\upsilon_{k}\left(g\right)=\frac{\left(1+\kappa_{k}\right)\left(1+\eta_{k}^{2}\right)}{2{\bar{\gamma }}_{k}}\left[1-\left(1-\frac{1}{\eta_{k}^{2}}\right)g^{2}\right]$$

and

(26) $$\Psi \left(a,b,y\right)=\frac{b\sqrt{\pi }}{2a^{5/2}}e^{\frac{b^{2}}{4a}}\left(1-e^{-ay}e^{b\sqrt{y}}\right)\left[\mathrm{erfc}\left(\frac{2a\sqrt{y}-b}{2\sqrt{a}}\right)+\mathrm{erfc}\left(\frac{b}{2\sqrt{a}}\right)\right],$$

with mean SINR for the *k*-th UE ${\bar{\gamma }}_{k}$ given by

(27) γ¯k=γqkμReαk2+σReαk2+σImαk2.

## 6. Performance Analyses

As described in Section 3, the LSF is assumed to remain constant for multiple transmitted frames. As a consequence, the derived metrics incorporate this characteristic of the system and is valid for $\tau_{l}$-long intervals.

### 6.1. Ergodic Capacity

In this study, the signal is transmitted over a frequency-non-selective and slow SSF channel. Hence, the normalized EC can be calculated by averaging the instantaneous capacity, conditioned to a given SINR γ, by the PDF of the channel SINR [35]. This assumption is consistent with the fact that the multiuser interference tends, by the LCLT, to a complex white Gaussian sequence that is added to the receiver’s white Gaussian noise. Thus, for the CF system, the *k*-th UE EC can be expressed by

(28) $${\bar{C}}_{k}=\int_{0}^{\infty }\log_{2}\left(1+y\right)p_{\gamma_{k}}\left(y\right)dy.$$

Substituting (18) into (28), and making use of the Gauss–Laguerre quadrature [36], one can write

(29) $${\bar{C}}_{k}=\frac{\left(1+\kappa_{k}\right)\left(1+\eta_{k}^{2}\right)}{2{\bar{\gamma }}_{k}N_{c}\eta_{k}\exp\left(p_{k}\right)}\sum_{c=1}^{N_{c}}\sum_{v=1}^{N_{v}}\frac{D_{v}T_{k}\left(y_{v},g_{c}\right)}{\upsilon_{k}\left(g_{c}\right)},$$

in which

(30) $$T_{k}\left(y,g\right)=\log_{2}\left(1+\frac{y}{\upsilon_{k}\left(g\right)}\right)\exp\left(r_{k}g\sqrt{\frac{y}{\upsilon_{k}\left(g\right)}}\right),$$

$D_{v}$ are the Gauss–Laguerre quadrature weights, given by

(31) $$D_{v}=\frac{y_{v}}{{\left(1+N_{v}\right)}^{2}{\left[L_{N_{v}+1}\left(y_{v}\right)\right]}^{2}}$$

and $y_{v}$ is the *v*-th root of the Laguerre polynomial of degree $N_{v}$.

Alternatively, a normalized channel capacity upper bound can be derived using Jensen’s inequality [37]. Hence,

(32) $${\tilde{C}}_{k}=\log_{2}\left(1+{\bar{\gamma }}_{k}\right),$$

in which ${\bar{\gamma }}_{k}$ is given by (27).

The normalized EC and normalized capacity upper bound per UE can be calculated, respectively, by

(33) $$\bar{C}=\frac{1}{K}\sum_{k=1}^{K}{\bar{C}}_{k}$$

and

(34) $$\tilde{C}=\frac{1}{K}\sum_{k=1}^{K}{\tilde{C}}_{k}.$$

### 6.2. Outage Probability

The OP of the *k*-th UE, that is, the probability of $\gamma_{k}$ to fall below the threshold SINR $\gamma_{\mathrm{th}}$, is given by

(35) $$P_{\gamma_{k}}\left(\gamma_{\mathrm{th}}\right)=\int_{0}^{\gamma_{\mathrm{th}}}p_{\gamma_{k}}\left(y\right)dy=P_{\gamma_{k}}\left(\gamma_{\mathrm{th}}\right).$$

Consequently, the per-UE OP can be calculated as

(36) $${\bar{P}}_{\gamma }\left(\gamma_{\mathrm{th}}\right)=\frac{1}{K}\sum_{k=1}^{K}P_{\gamma_{k}}\left(\gamma_{\mathrm{th}}\right).$$

## 7. Simulation Setup

The UC CF system is adopted with an AP selection strategy based on the SINR expression with $\#M_{k}=5$, similarly to the one presented in [24]. It is considered that $\tau_{l}=40$ frames of $\tau_{s}=200$ symbols are sent per scenario realization. The adopted operation frequency is 28 GHz and the path loss coefficients are found in [38] and, without loss of generality, uniform power allocation is assumed [2]. For the estimation phase, $\tau_{p}=20$ with $\rho_{p}=0.5\rho$ is adopted.

The LoS components occur according to a distance dependence probability model [39]. Thus, one may observe links with or without LoS components between UEs and APs. Therefore, if $A_{mk}$ is a Bernoulli random variable, whose success indicates the presence of an LoS component in the AP-to-UE link, one can write [5]

(37) $$\mathrm{Prob}\left\{\left.A_{mk}=1\right|d_{mk}\right\}=p_{\mathrm{LoS}}\left(d_{mk}\right),$$

in which $d_{mk}$ denotes the distance between the *m*-th AP and the *k*-th UE, and [39]

(38) $$p_{\mathrm{LoS}}\left(d\right)=\min\left(\frac{19.1}{d},1\right)\left(1-e^{-\frac{d}{18.3}}\right)+e^{-\frac{d}{18.3}},$$

with $\min\left(x,y\right)$ denoting the minimum between *x* and *y*.

Similarly to the LSF coefficient, the ${\bar{h}}_{mk}$ component is assumed to remain constant throughout $\tau_{l}$ transmitted frames. Additionally, to guarantee the system energy conservation, $\beta_{mk}$ must be adjusted due to the presence of the LoS component. Hence, one can write [5]

(39) $$\beta_{mk}=\frac{10^{\frac{P_{mk}}{10}}S_{mk}}{1+A_{mk}\nu_{mk}},$$

in which $S_{mk}$ is the log-normal shadowing, $P_{mk}$ is the ABG path loss and

(40) $$\begin{array}{c} \nu_{mk}=10^{1.3-0.003d_{mk}} \end{array}$$

is the Rice factor [5].

Finally, the AP-to-UE LoS component can be expressed by [5]

(41) $${\bar{h}}_{mk}=A_{mk}\sqrt{\nu_{mk}}e^{j\theta_{mk}},$$

in which $\theta_{mk}$ is a random variable with uniform distribution, $\theta_{mk}∼U\left[-\pi ,\pi \right]$.

Notice that, when $A_{mk}=0$, no LoS component is observed in that AP-to-UE link.

## 8. Results

**Figure 2:**
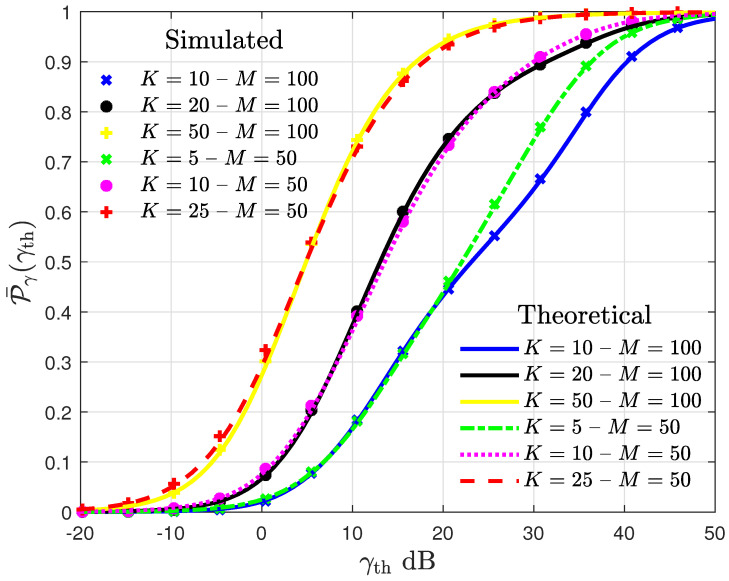
Curves of OP as a function of $\gamma_{\mathrm{th}}$ for different values of *M* and *K* assuming #M=5 and ρ=0.5 W.

The curves of OP in Figure 2 reveal a good agreement between the simulated data and the theoretical results. It is observed that, in general, keeping the ratio of UE per AP, the OP curves present a similar behavior. However, as the scenario sparsity, in terms of UEs, and the number of APs increase, it leads to $M_{k}$ sets that tend to differ from UE to UE. These disjoint $M_{k}$ sets, in addition to the higher level of spatial macro-diversity, imposed by a higher value of *M*, allow the AP selection algorithm to exploit the spatial macro-diversity and increase the SINR of some UEs, improving the system performance, as observed in the missing agreement of the curves for M=50 with K=5 and M=100 with K=10 for $\gamma_{\mathrm{th}}>20$ dB. Furthermore, it is observed that, for a fixed number of APs, the system performance worsens as the number of UEs increases. For instance, for $\gamma_{\mathrm{th}}=20$ dB and considering M=100 APs, the OP goes from ≈43% to ≈73% as *K* increases from 10 to 20 UEs.

**Figure 3:**
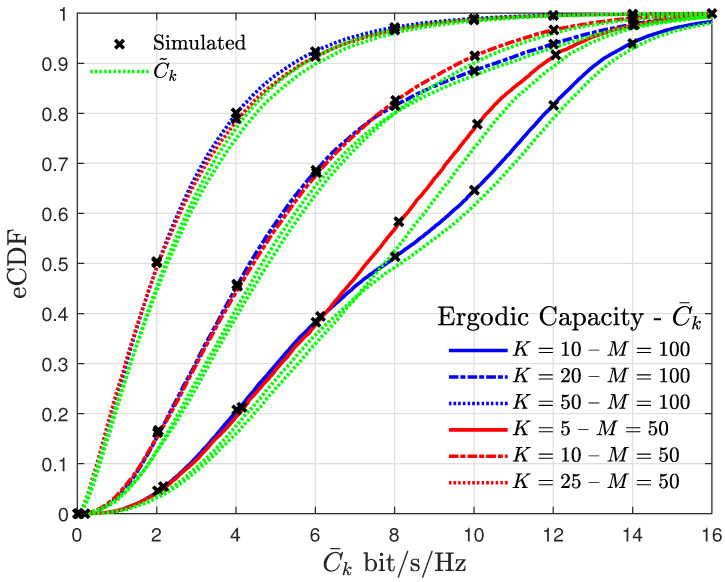
Curves of the eCDF of the UE EC for different values of *M* and *K* assuming #M=5 and ρ=0.5 W.

Curves of eCDF of the normalized UE EC are presented in Figure 3, for different *M* and *K*. The curves obtained using (32) are system performance upper bounds and are useful for power allocation strategies. However, those results do not reflect the real metric value, leading to a system performance overestimation. Hence, compared to the EC given by (29), the results obtained using Jensen’s inequality are less appropriate for algorithms that aim to maximize channel usage.

A similar pattern is found in the curves for M=100 with K=10 and M=50 with K=5 in the EC results, for ${\bar{C}}_{k}>6$ bits/s/Hz, and in the OP results, for $\gamma_{\mathrm{th}}>20$ dB. An inflection point indicates the rising of multimodal distributions, characterized by different SINR regions with higher probability. In such situations, an optimization algorithm for UE quality-of-service (QoS) fairness can be applied to provide uniform rates to the UEs.

**Figure 4:**
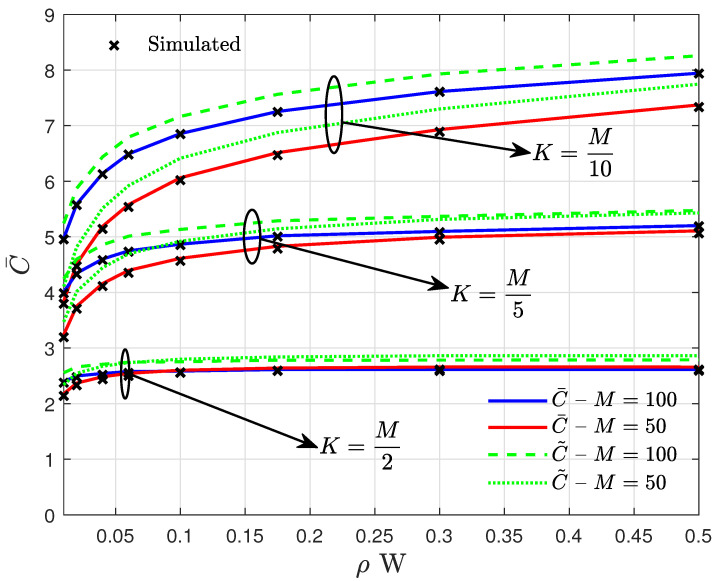
Curves of the EC per UE for different values of *M* and *K* assuming #M=5.

The saturation trends observed in the curves of $\bar{C}$ and $\tilde{C}$, presented in Figure 4, appear as a result of increasing the multiuser interference power $\sigma_{\psi_{k}}^{2}$ with ρ. However, as the ratio of UEs per APs increases, the probability of disjoint $M_{k}$ sets decreases, increasing the average number of interfering signals experienced by each UE, leading to a faster performance saturation, as observed for M=50 with K=25 and M=100 with K=50. It is worth mentioning that, although well accepted, the results obtained by Jensen’s inequality, given by (32), may present a limited application. That is observed, for instance, in the curves for M=50 and K=25 with errors up to 10%, contrasting with the 1.5% errors reached by the EC results given by (29).

**Figure 5:**
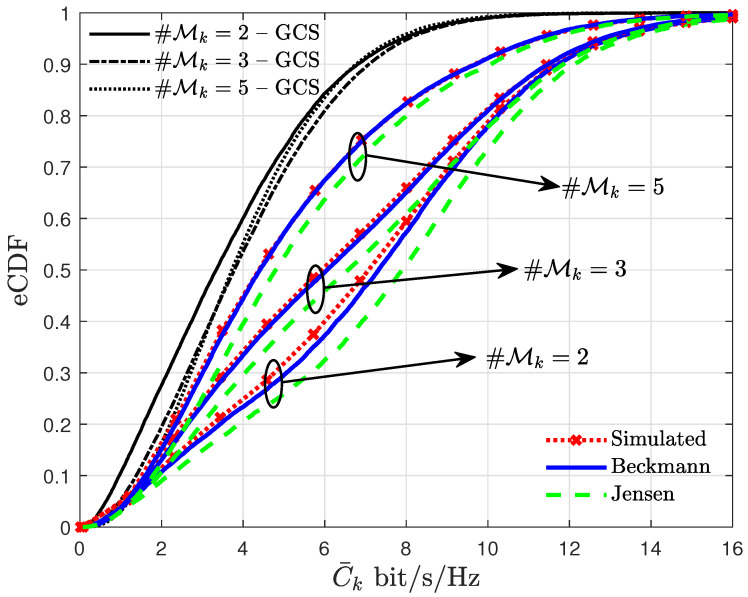
Curves of the eCDF of the UE EC for different values of $M_{k}$ considering K=10, M=50 and assuming ρ=0.5 W.

Curves of eCDF generated from EC expressions calculated for GCS and Beckmann effective channel gain models are presented in Figure 5 and compared with the upper bound provided by Jensen’s inequality. For the results in Figure 5, $\rho_{p}>>\rho$ is assumed, so that the estimation error can be neglected. It is observed that, when the scenario exhibits AP-to-UE links in LoS, the EC expression derived taking the GCS as the effective gain distribution fails to predict the per-UE EC. On the other hand, for $\varepsilon_{mk}\to 0$, the envelope of the effective channel gain degenerates to the modulus of a Gaussian random variable, which is a special case of the Beckmann distribution. Thus, the results obtained using (29) are able to predict the simulated data with a good agreement, in contrast with the results obtained considering the GCS distribution. Furthermore, the improvement achieved by applying an SINR-based AP selection technique is also perceived if a proper channel characterization is used. When Beckman distribution is considered, one can observe that $\mathrm{Prob}\left\{{\bar{C}}_{k}>6\;\mathrm{bit}/s/\mathrm{Hz}\right\}$ increases from ≈34% to ≈63% when $M_{k}$ decreases from 5 to 2. On the other hand, the obtained gain for GCS fading model is approximately only 4%.

The rise in inflection points, similar to the ones observed in Figure 2 and Figure 3 for K=10 and M=100, observed in Figure 5 for K=10 and M=50, may be justified with the increasing probability of disjoint $M_{k}$ sets as the numbers of assigned APs to each UE decrease. Additionally, one can notice that increasing $\#M_{k}$ leads to a worse system performance, which is justified by the growth of $\sigma_{\psi_{k}}^{2}$ as predicted by (17).

## 9. Conclusions

In this work, we proposed applying the Lyapunov Central Limit Theorem to approximate the multiuser interference and the effective channel gain in a user-centric cell-free downlink network by complex Gaussian random variables. Therefore, the envelope of the effective channel gain can be characterized by the Beckmann probability distribution, and the multiuser interference, together with the additive white Gaussian noise, compose a single additive Gaussian noise. From this proposed model of downlink-received signal, simple expressions are derived to evaluate the outage probability, the normalized ergodic capacity and the maximum transmission rate by means of the Jensen inequality. Although widely accepted in the literature, the maximum transmission rate provides lower accuracy when compared to EC. The curves obtained from the derived OP and EC expressions adhere closely to the simulated data. In contrast, the EC upper bound curve plotted from the Jensen’s inequality-based expression presents a considerable distance from the simulated data. Aside from comparing derived expressions with simulated data, this paper discusses the faults by inappropriately modeling the effective channel gain by the GCS distribution in scenarios with links in LoS, which leads to inaccurate metric value prediction that may reduce, for example, the effectiveness of AP selection algorithms.

## Data Availability

The data presented in this study are available on request from the corresponding author.

## Abbreviations

The following abbreviations are used in this paper:

CF	Cell-Free
mMIMO	massive Multiple-Input-Multiple-Output
AP	Access Point
UE	User Equipment
MMSE	Minimum Mean-Squared-Error
CPU	Central Processing Unit
GCS	Generalized Chi-Squared
CSI	Channel State Information
LoS	Line-of-Sight
AR	Achievable Rate
UC	User-Centric
LSF	Large-Scale Fading
SINR	Signal-to-Interference-plus-Noise-Ratio
MRT	Maximum-Ratio Transmission
NLoS	Non-Line-of-Sight
EC	Ergodic Capacity
OP	Outage Probability
LCLT	Lyapunov Central Limit Theorem
SSF	Small-Scale Fading
CDF	Cumulative Density Function
RF	Radio Frequency
VLC	Visible Light Communication
ASR	Achievable Sum Rate
JUAP	Joint UE and AP Preference-based Associated Scheme
RSRP	Reference Signal Received Power
RU	Radio Unit
eCDF	empirical Cumulative Density Function
RSS	Received Signal Strength
V2X	Vehicle to Everything
DRL	Deep Reinforcement Learning
RIS	Reconfigurable Inteligent Surface
TDD	Time Division Duplex
ABG	Alpha–Beta–Gamma
INID	Independent and Non-Identically Distributed
